# New Shoes Made Easy

**Published:** 1854-03

**Authors:** 


### NEW SHOES MADE EASY.

The luxury of an "old shoe" is universally appreciated. Any one so fortunate as to be able to prefer comfort to looks, parts with an old boot or shoe with great reluctance. The money cost of a new pair may be of no consequence, but the suffering which they will in all probability occasion for days, if not weeks, cannot be lightly regarded. Corns are the necessary result of a tight shoe, giving discomfort, pain, and sometimes torture for the remainder of life. Every physician knows that corns improperly tampered with, sometimes destroy life, the sufferings from them in such cases being often terrible—literally, terrible.

The Editor, for some years past, has had no trouble in having a new boot or shoe made to feel easy and comfortable, not only for the first week or two, but from the time they are first put on, until they are worn out: and, for the sake of "toes" proposes the expedient for trial, thinking that it may work as well with others as it has always done with himself.—Before going to have your measure taken, or to select a pair already made, put on two pair of thick woollen socks.

The expedient is simple, easily tried, and has given to the Editor results, really delightful.

